# Neural Correlates of Moral Evaluation and Psychopathic Traits in Male Multi-Problem Young Adults

**DOI:** 10.3389/fpsyt.2018.00248

**Published:** 2018-06-11

**Authors:** Josjan Zijlmans, Reshmi Marhe, Floor Bevaart, Marie-Jolette A. Luijks, Laura van Duin, Henning Tiemeier, Arne Popma

**Affiliations:** ^1^Department of Child and Adolescent Psychiatry, VU University Medical Center, Amsterdam, Netherlands; ^2^Department of Social and Behavioral Sciences, Harvard T.H. Chan School of Public Health, Boston, MA, United States; ^3^Department of Child and Adolescent Psychiatry, Erasmus Medical Center, Rotterdam, Netherlands; ^4^Department of Epidemiology, Erasmus Medical Center, Rotterdam, Netherlands; ^5^Department of Criminal Law and Criminology, Leiden University, Leiden, Netherlands

**Keywords:** psychopathy, morality, fMRI, young adulthood, amygdala, ventromedial prefrontal cortex

## Abstract

Multi-problem young adults (18–27 years) present with a plethora of problems, including varying degrees of psychopathic traits. The amygdala and ventromedial prefrontal cortex (vmPFC) have been implicated in moral dysfunction in psychopathy in adolescents and adults, but no studies have been performed in populations in the transitional period to adulthood. We tested in multi-problem young adults the hypothesis that psychopathic traits are related to amygdala and vmPFC activity during moral evaluation. Additionally, we explored the relation between psychopathic traits and other regions consistently implicated in moral evaluation. Our final sample consisted of 100 multi-problem young adults and 22 healthy controls. During fMRI scanning, participants judged whether pictures showed a moral violation on a 1–4 scale. Whole brain analysis revealed neural correlates of moral evaluation consistent with the literature. Region of interest analyses revealed positive associations between the affective callous-unemotional dimension of psychopathy and activation in the left vmPFC, left superior temporal gyrus, and left cingulate. Our results are consistent with altered vmPFC function during moral evaluation in psychopathy, but we did not find evidence for amygdala involvement. Our findings indicate the affective callous-unemotional trait of psychopathy may be related to widespread altered activation patterns during moral evaluation in multi-problem young adults.

## Introduction

Psychopathy is a psychological construct characterized by affective callous-unemotional traits, impulsive and irresponsible behavior, and grandiose-manipulative interpersonal traits (1). Individuals with high psychopathic traits engage in morally inappropriate behavior (e.g., committing crimes, lying, and cheating) and show a lack of guilt or remorse after performing antisocial actions (2). However, research suggests they are generally capable of differentiating right from wrong when evaluating moral dilemmas or situations (3, 4) and their moral reasoning can thus prove normal when assessed behaviorally. In the last decade, there has been an increasing interest in the underlying neurobiology of moral reasoning. Clarifying which neural processes are aberrant in persons with psychopathic traits may help understand why individuals with high psychopathic traits engage in their immoral behaviors.

Previous studies have investigated the relationship between psychopathic traits and moral processing in (forensic) adolescents and adults, but as of yet no research has specifically focused on a young adult group (aged 18–27). Given that the transition from adolescence to adulthood (5) is especially challenging for vulnerable populations (6) young adulthood is an important period to study distinctly (7). In the present study, we investigated a sample of young adults (18–27 years old) who dysfunction in society and suffer from multiple problems. These multi-problem young adults lack a stable income, do not have the prerequisites to get a job, most of them have engaged in criminal activities of ranging seriousness (e.g., from shoplifting to violent crimes), and two thirds of them have had Child Protection Service (CPS) interference, chiefly due to judicial problems before age 18 (8). It is an ecologically valid sample in which antisocial behavior is displayed in varying degrees. Therefore, we expect their psychopathic traits to vary accordingly from very low to very high. We measured psychopathy continuously, which is preferable over a taxonomic approach (9–11) as it allows for the entire range of the construct to be taken into account.

Brain areas that have been implicated in the processing of moral information in healthy populations include the amygdala and ventromedial prefrontal cortex (vmPFC), which are important for processing emotional information; the dorsolateral prefrontal cortex (dlPFC), which is engaged in utilitarian decision making; and the superior temporal gyrus (STG), which is important when thinking about others (4, 12–15). A recent meta-analysis of fMRI research of moral processing (16) distinguished between studies investigating moral judgments about situations (moral evaluations) and studies requiring participants to make moral decisions as if they were the actor (moral response decisions), as evidence suggests these are at least partially different processes (17). The current study focuses on moral evaluations. Six brain areas were found to be specifically involved in making moral evaluations (16): the right and left STG, the left cingulate gyrus (CG), the right medial frontal gyrus (MFG; part of the vmPFC) and two distinct areas in the left MFG (one in BA9, one in BA10).

It has been argued that amygdala and vmPFC dysfunction lie at the basis of the moral impairments of individuals high in psychopathic traits (18). The amygdala is suggested to aversively reinforce actions that harm others; the vmPFC in turn processes this information and creates an outcome expectancy. When amygdala functioning is disrupted, the vmPFC fails to appropriately represent the valenced information, leading to immoral behavior. To date, decreased amygdala activity during moral processing (immoral minus nonmoral contrasts, where nonmoral stimuli are negative but not immoral) has been found in community volunteers with psychopathic traits (19), but not in incarcerated males (20), incarcerated adolescents (21), or incarcerated women (22). However, in the adolescent and female samples negative correlations between psychopathic traits and amygdaloid brain activity have been observed when contrasting both immoral and nonmoral stimuli with neutral stimuli (which are neither negative nor immoral). Negative correlations between vmPFC activity and psychopathic traits during moral processing have been reported in some studies (19, 20), but not others (21, 22). In short, although a theoretical basis for amygdala and vmPFC dysfunction underlying moral dysfunction in psychopathy exists (18), neuroimaging results vary depending on samples and different ranges and variation of psychopathic traits within these samples.

As mentioned, in the current study we investigated a sample of multi-problem young adults (18–27 years old) with varying levels of psychopathic traits. At the time of assessment, all participants were enrolled in a day treatment program intended to help reintegrate into society. Within this heterogeneous sample, we employed a task that requires participants to make moral evaluations about situations that are presented as pictures. We included a group of healthy controls primarily to assess whether the task worked appropriately and whether multi-problem young adults are capable of performing the task. We expected to find brain activity related to moral evaluation in line with the current literature (i.e., increased vmPFC, STG, and CG activity), and tested the hypothesis that the three psychopathic dimensions are negatively related to amygdala and vmPFC activity during moral evaluation in multi-problem young adults. We also tested whether psychopathic traits are associated with other regions consistently implicated in moral processing (i.e., STG and CG). We expected the affective callous-unemotional dimension of psychopathy to be specifically relevant as it is representative of behavior related to moral evaluation (i.e., shallow affect, lack of empathy, lack of remorse). We investigated the impulsive-irresponsible and grandiose-manipulative dimension exploratively.

## Methods

### Participants

Participants were 110 male multi-problem young adults [part of a larger study including 696 multi-problem young adults; (23)]. They were recruited at the start of day treatment program *De Nieuwe Kans* (DNK; translated as “New Opportunities”). DNK provides a multimodal day treatment program, which aims to increase the self-sufficiency and decrease recidivism of multi-problem young adults. Additionally, 25 age and gender group matched healthy controls were included in the study. Controls were selected to have average education. Exclusion criteria for the fMRI study were non-corrected defective vision and fMRI contra-indications. Ten multi-problem young adults were excluded due to excessive movement (*N* = 3) or poor task performance (e.g., more than 5 missed trials; *N* = 7). Three controls were excluded due to poor task performance. The final sample included 100 multi-problem young adults and 22 healthy controls. See Table 1 for an overview of the descriptive data.

**Table 1:** Participant characteristics.

	**Multi-problem young adults (*****N*** = **100)**		**Healthy controls (*****N*** = **22)**		
	***M* (range)**	***SD***	***M* (range)**	***SD***	***p***
Age	22.56	2.41	23.19	2.84	0.28
IQ	82.98 (60–107)	10.65	–	–	–
YPI-SV total	34.08 (19–68)	7.92	35.5 (25–50)	5.28	0.42
YPI-SV affective callous-unemotional	10.68 (6–24)	3.53	11.53 (8–16)	2.57	0.29
YPI-SV impulsive-irresponsible behavioral	12.19 (7–21)	3.1	12.05 (8–18)	2.57	0.84
YPI-SV grandiose manipulative interpersonal	11.21 (6–23)	3.71	11.95 (6–19)	3.23	0.39
Cannabis use past 30 days	14.80 (0–30)	13.24	4.18 (0–16)	6.40	< 0.001
Years of regular cannabis use	4.34 (0–14)	3.78	1.30 (0–10)	2.60	< 0.001
**RATINGS OF STIMULI**					
Neutral	1.12	0.19	1.07	0.16	0.25
Nonmoral	2.17	0.65	1.86	0.64	0.04
Immoral	3.21	0.41	3.41	0.4	0.04
**EDUCATION**					
No secondary education	90%	–	0%	–	–
Secondary education following	0%	–	41%	–	–
Secondary education finished	10%	–	59%	–	–

This study was carried out in accordance with the recommendations of the Medical Ethical Committee of the VU University Medical Center. The protocol was approved by the VU University Medical Center Medical Ethical Committee (registration number 2013.422 - NL46906.029.13). All subjects gave written informed consent in accordance with the Declaration of Helsinki. Participants received a reimbursement of 30 euros for their participation in the fMRI protocol and an EEG protocol, which was administered on another day.

### Instruments

Psychopathic traits were assessed using the Youth Psychopathy Inventory – Short Version [(24); also validated in young adults; (25)]. The YPI-SV is a self-report measure that distinguishes three factors of psychopathy: an affective callous-unemotional factor, a behavioral impulsive-irresponsible factor, and an interpersonal grandiose-manipulative factor. We used the Measurements in the Addictions for Triage and Evaluation Questionnaire (MATE) to assess current and historic drug use. In order to measure intelligence, we used the short form of the Wechsler Adult Intelligence Scale third version (WAIS-III SF) consisting of four subtests (26): digit symbol coding, information, block design, and arithmetic. The WAIS-III-SF was only assessed in the multi-problem group. See Table 1 for descriptive data.

### Stimuli

Three types of stimuli were used in the experiment: 25 immoral and negative (e.g., a person threatening another person with a knife); 25 non-moral and negative (e.g., people shouting at each other); and 25 neutral (e.g., people sitting next to each other). In order to select the stimuli, we first presented a set of 120 stimuli selected from the International Affective Picture System (IAPS; 27) (40 of each type as assessed by JZ) to a pilot group of 134 participants via the online tool Mechanical Turk (28) and asked the participants to rate them on a 1-7 scale on their morality, valence, arousal, and complexity. From each category, 25 pictures were chosen in such a way that the immoral and nonmoral pictures matched on valence, arousal, and complexity, but not on morality. In other words, the moral and non-moral stimuli are specifically distinguishable on morality, which allows us to disentangle moral transgression from negative valence. For an overview of the average ratings of the stimuli see Table 2.

**Table 2:** Average ratings of stimuli via Mechanical Turk (*N* = 134).

	**Moral violation**	**Valence**	**Arousal**	**Complexity**
Immoral	5.50	1.68	5.11	4.36
Nonmoral	1.94	1.94	4.90	4.13
Neutral	1.03	4.24	2.52	2.45

### Procedure and task

Participants were told they would be shown pictures that might contain moral violations and were instructed to judge whether this was the case or not on a scale from 1 to 4, where 1 indicated that no moral violation was presented in the picture and 4 indicated that a major moral violation was presented in the picture. Participants were asked to consider the morality of the event depicted whilst viewing the picture and to press on the appropriate button after the picture was replaced by a rating scale. Participants were told that there were no right or wrong answers and that we were interested in their personal opinion.

Each trial consisted of a picture being shown for 5 s, a rating scale of 1–4 being shown for 3 s, and a variable inter stimulus interval of 3–8 s (average 5.5 s), introducing jitter into the experimental design. Pictures were presented pseudo randomly, with a maximum of three pictures of the same condition being shown sequentially. Participants performed three practice trials in order to ensure they understood the experiment. The experiment was administered in two sessions (the first with 38 stimuli, the second with 37 stimuli) with a small break in between so that the task would not be too demanding. The task is based on and similar to that used by Harenski et al. (20). We adopted a different response procedure, requiring participants to press one of four buttons whenever they had decided on their answer, rather than pressing one button when the answer they wanted to give was shown on the screen. Also, we introduced jitter by varying the intertrial intervals around a mean value, rather than using three distinct intertrial intervals. The experiment was created and presented with Presentation 17.1 (https://www.neurobs.com/).

### MRI data acquisition and analysis

MRI data were collected on a 3T GE Healthcare MRI scanner at Erasmus Medical Center Rotterdam. Structural T1-weighted images were acquired with a fast-spoiled gradient pulse sequence in 180 sequential sagittal slices, with a thickness of 1.0 mm. The repetition time (TR) was 6.4 ms, the echo time (TE) 2.8 ms, the flip angle (FA) 12°, the field of view (FOV) 240 mm, and the matrix size 240 × 240 mm. Blood oxygen level-dependent T2^*^-weighted images were acquired axially with an echo planar imaging gradient echo pulse sequence in 42 slices of 3.5 mm with a slice spacing of 0.5 mm. The TR was 2,000 ms, the TE 30 ms, the FA 80°, the FOV 220 mm, and the matrix size 64 × 64 mm.

Functional imaging data were analyzed using Statistical Parametric Mapping 12 (SPM12; http://www.fil.ion.ucl.ac.uk/spm/). As preprocessing steps, for each participant functional images were realigned and unwarped, the structural scan was segmented and co-registered to the mean T2^*^-weighted image. Images were then normalized to the MNI template and smoothed with an 8mm full-width half maximum Gaussian filter. The three conditions were modeled as 5-s events with the standard hemodynamic response function. Six movement parameters were added as covariates of no interest. Our contrast of interest was the immoral > nonmoral contrast, which is expected to be representative of brain activity due to the moral salience of the stimuli, controlled for the (negative) emotional valence of the stimuli.

We defined a priori regions of interest (ROI) based on the most recent meta-analysis concerning the neural correlates of moral decision-making (16). We limited the ROIs to areas that were found to be consistently active during moral evaluation tasks. The a priori ROIs are the right and left superior temporal gyrus (STG;), the left cingulate gyrus (CG), the right vmPFC, and two distinct areas in the left vmPFC (one in BA9, one in BA10). Additionally, we included the right and left amygdala as ROIs since the amygdala has been implicated both theoretically (18) and empirically (19, 29) to be involved in moral processing and psychopathy. ROIs were created by forming 8mm-radius spheres around the peak coordinates (r-STG 53 12-28; l-STG-45-57 16; l-CG-3-59 26; r-vmPFC 3 59 4; l-vmPFC (BA9)-6 47 23; l-vmPFC (BA10)-4 54-5; l-amygdala-22-6-16; r-amygdala 24-4-14) that are reported in the literature (16, 29). For the creation of the ROIs and the extraction of the ROI data we used the Marsbar toolbox for SPM (http://marsbar.sourceforge.net/).

For the behavioral and questionnaire data, in order to compare the ratings of the three picture types between each other and between the multi-problem group and the control group, we performed a repeated measures ANOVA with group as between subjects factor and *post-hoc t*-tests. To investigate the relationship with IQ in the multi-problem group we performed Pearson's correlations between psychopathy, IQ, and behavioral responses. All thresholds were set to α = 0.05 and *post-hoc* tests were Bonferroni corrected.

For the neuroimaging data, we performed whole-brain analysis in SPM12. The conditions were modeled in the context of the general linear model, using the canonical hemodynamic response function. Standard family-wise error correction (*p* < 0.05) was applied. For the association between psychopathy and ROI-activity during moral evaluation in the multi-problem group, we performed multiple linear regression analyses using the total psychopathy score or psychopathy subscale scores as independent variables and the extracted ROI data as dependent variables. We performed the analyses with and without IQ and drug use as covariates. Cannabis was the only drug prevalent enough in our sample to take into account (see Table 1), we controlled for both recent cannabis use (past 30 days) and historic cannabis use (amount of years that the drug was used at least once per week).

## Results

### Behavioral results

We compared the ratings of the three picture types between each other and between groups. A repeated measures ANOVA with picture type as within subjects factor and group as between subjects factor showed that the three types of pictures were rated differently in terms of moral violations across all participants [*F*_(2, 240)_ = 530.52, *p* < 0.001]. Immoral pictures (*M* = 3.25, *SD* = 0.42) were rated as greater moral violations than nonmoral pictures [*M* = 2.11, *SD* = 0.65; *t*_(121)_ = 18.02, *p* < 0.001] and neutral pictures [*M* = 1.1, *SD* = 0.19; *t*_(121)_ = 52.34, *p* < 0.001]. Nonmoral pictures were rated as greater violations than neutral pictures [*M* = 1.11, *SD* = 0.19; *t*_(121)_ = 18.14, *p* < 0.001]. There was no main effect of group (*p* > 0.05), but we found a significant interaction between picture type and group [*F*_(6.84, 240)_ = 6.84, *p* < 0.001].

Multi-problem young adults judged immoral pictures to represent smaller violations (*M* = 3.21, *SD* = 0.41) than controls did [*M* = 3.41, *SD* = 0.40; *t*_(120)_ = −2.10, *p* < 0.05]. In contrast, multi-problem young adults judged nonmoral pictures to represent larger violations (*M* = 2.17, *SD* = 0.65) than controls did [*M* = 1.86, *SD* = 0.64; *t*_(120)_ = 2.00, *p* < 0.05]. However, after Bonferroni correction, these tests failed to reach significance. Both groups rated the neutral pictures similarly (*p* > 0.05). See Table 1 for an overview.

Within the multi-problem group, none of the ratings of picture types were significantly related to total psychopathy score and psychopathic traits (all *p*s > 0.05). We found a negative correlation between IQ and rating of the nonmoral stimuli (*r* = −0.41, *p* < 0.001) thus multi-problem young adults with lower IQs judged nonmoral stimuli to show larger moral violations. IQ was not significantly related to the ratings of immoral (*r* = 0.08, *p* = 0.41) and neutral stimuli (*r* = −0.17, *p* = 0.11). Finally, IQ was not significantly related to any psychopathic traits (all *p*s > 0.05).

### Imaging results

**Figure 1:**
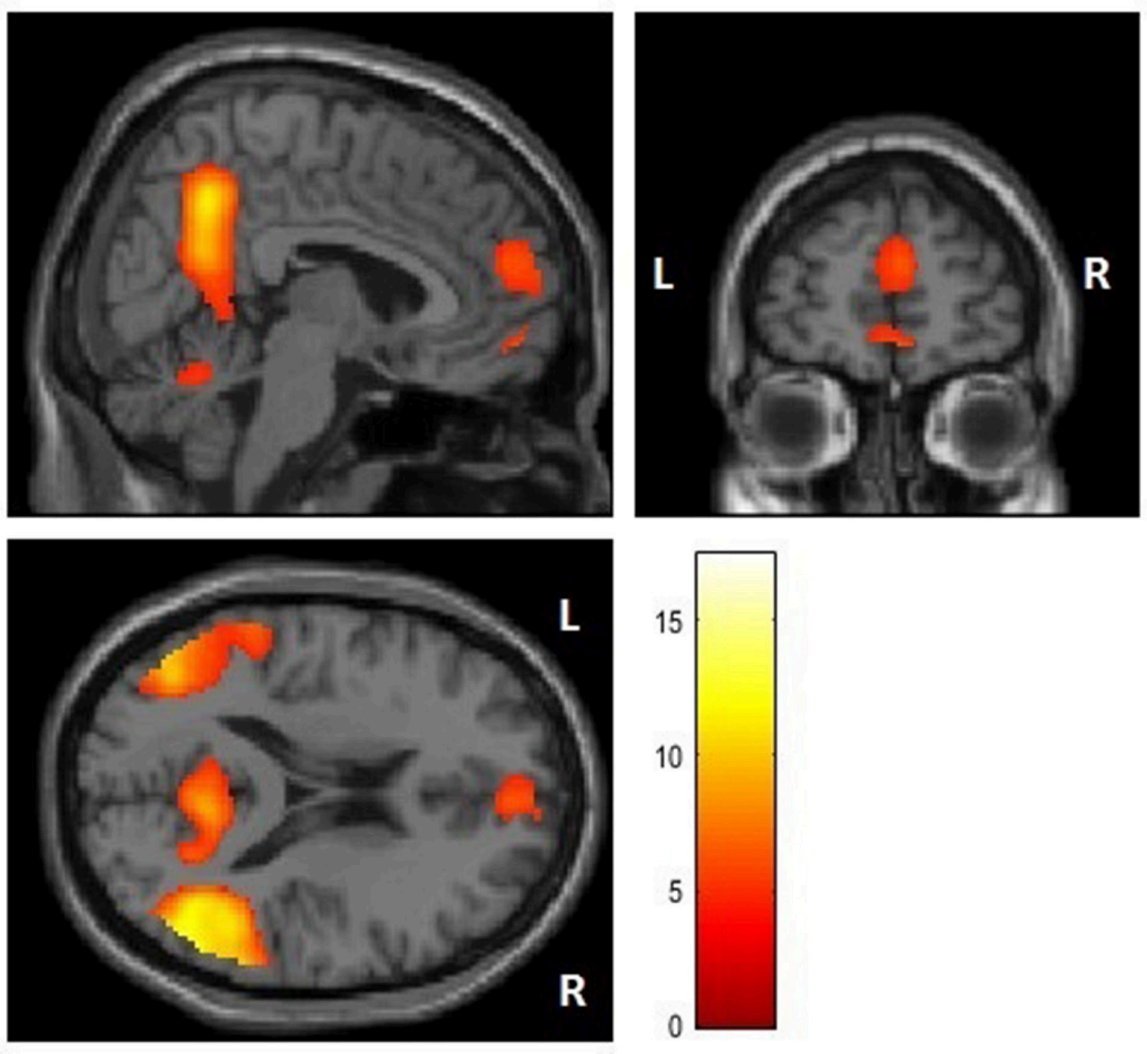
Whole brain analysis of the immoral > nonmoral contrast (x = 6, y = 56, z = 19).

First, we performed a whole brain analysis of the contrast of interest moral > nonmoral across all participants. This revealed significant increased hemodynamic responses in the STG, in several distinct areas of the vmPFC, in the precuneus, the parahippocampal gyrus, in the middle occipital gyrus (MOG), and in the cerebellum (see Figure 1). Also, we found increased activation in the precentral gyrus and the thalamus, this is likely due to the setup of the task: when participants evaluated pictures as not representative of a moral violation or as representative of a slight moral violation they used their left hand, when participants evaluated pictures as representative of a somewhat immoral violation or a strong moral violation participants used their right hand (see Table 3 for an overview). No significant differences in activation patterns in any of the brain regions were found between the experimental group and the control group in the analyses of this contrast.

**Table 3:** fMRI whole brain analysis (immoral > nonmoral), *N* = 122.

**Cluster-level**		**Peak-level**					
**p(FWE-corr)**	***k***	**p(FWE-corr)**	***T***	**x {mm}**	**y {mm}**	**z {mm}**	**Location of peak coordinate**
0.000	2,856	–	17.41	48	−58	12	STG
0.000	2,310	–	17.10	−48	−70	6	MOG
		0.000	7.34	−58	−38	24	
0.000	3,394	–	11.48	4	−56	40	Precuneus
		–	9.70	4	−56	26	
		0.000	7.93	20	−54	14	
0.000	549	–	9.69	18	−52	−24	Cerebellum
0.001	157	0.000	7.15	22	−32	−18	Parahippocampal gyrus
		0.002	5.43	28	−20	−18	
0.004	79	0.000	6.22	16	−62	−48	Cerebellum
0.000	495	0.000	6.17	4	56	20	vmPFC
		0.000	6.08	4	56	−10	
		0.000	5.74	4	64	14	
0.008	48	0.002	5.41	40	4	44	vmPFC
0.014	26	0.006	5.06	26	26	42	vmPFC

**Figure 2:**
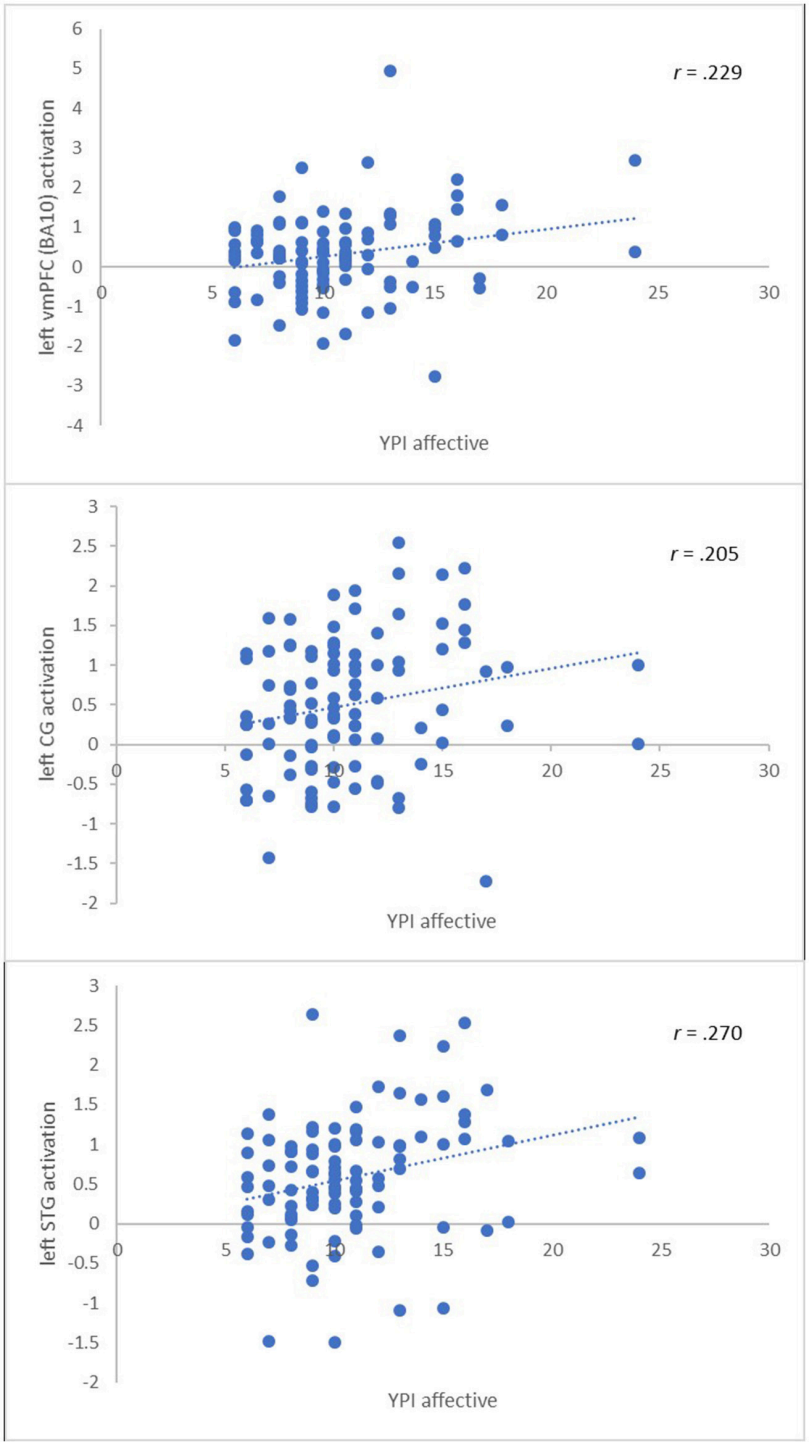
Significant associations between the callous-unemotional affective trait of psychopathy and brain activity in a priori ROIs.

Second, we extracted the summary time courses for the a priori defined ROIs and performed linear regression analyses to examine the relationship between the level of psychopathy and brain activity of the immoral > nonmoral contrast in the multi-problem group. We found the total psychopathy score to be positively related to brain activation in the left vmPFC (BA10) (β = 0.22, *p* < 0.05) and the left STG (β = 0.23, *p* < 0.05). We found no significant relation in the other regions of interest. We performed hierarchical regression analyses adding one subscale of the YPI at each level in order to assess their individual contributions to the models. We found the affective callous-unemotional factor score of psychopathy to be positively related to brain activation in the left vmPFC (BA10) (β = 0.23, *p* < 0.05), left STG (β = 0.27, *p* < 0.01), and CG (β = 0.21, *p* < 0.05), see Figure 2. The behavioral and interpersonal factors did not predict brain activity in any of the ROIs. See Table 4 for an overview.

**Table 4:** Regression analyses on extracted a priori ROI summary time courses, *N* = 100.

**Predictor**	**Outcome**	***R*^2^**	***p***
Total psychopathy score	vmPFC L (BA10)	0.048	0.028
	CG L	0.029	0.092
	STG L	0.052	0.022
	vmPFC R	0.028	0.099
	vmPFC L (BA9)	0.011	0.300
	STG R	0.009	0.347
	Amygdala L	0.006	0.461
	Amygdala R	0.000	0.910
Affective callous-unemotional score	vmPFC L (BA10)	0.053	0.022
	CG L	0.042	0.041
	STG L	0.073	0.007
	vmPFC R	0.036	0.057
	vmPFC L (BA9)	0.012	0.287
	STG R	0.009	0.354
	Amygdala L	0.004	0.552
	Amygdala R	0.000	0.862

*The behavioral and interpersonal traits of psychopathy were not significantly related to brain activity in the a priori ROIs*.

Third, we added IQ and drug use as covariates in the models. This did not change any of the reported results, but IQ itself was positively related to brain activity in the left vmPFC (BA10; β = 0.18, *p* < 0.05), left STG (β = 0.20, *p* < 0.05), and left CG (β = 0.20, *p* < 0.05).

## Discussion

In this study, we investigated the relationship between psychopathic traits and the neural correlates of moral evaluation in a sample of male multi-problem young adults. Multi-problem young adults were able to discriminate between immoral and nonmoral stimuli, but less well so than the control group, as they gave nonmoral pictures higher moral ratings and immoral pictures lower ratings. In line with previous research, several specific brain regions showed increased activity during moral evaluation, most notably the vmPFC, STG, parahippocampal gyrus, and precuneus. We did not find any negative relationships between the brain activity in these regions and psychopathic traits in the amygdala and vmPFC. Rather, we found no effect in the amygdala and found positive associations in the left vmPFC, left STG, and left CG, indicating that multi-problem young adults high in psychopathic traits show a greater increase in brain activity in these areas during moral evaluation than multi-problem young adults low in psychopathic traits.

Multi-problem young adults discriminated less between immoral and nonmoral stimuli than the control group. This difference does not seem to be driven by psychopathic traits, as both groups have similar psychopathic trait scores, and we found no relation between psychopathic traits and participants' responses to the stimuli. Potentially, this finding might be due to a difference in intelligence between groups. Although we did not formally assess the IQ of the control group, we can assume they have a higher level of intelligence than the multi-problem young adults given the low average IQ (mean IQ 83) and lower educational level of the multi-problem young adults. Furthermore, within the multi-problem group there was a moderately strong and negative correlation (*r* = −0.41) between IQ and the rating of the nonmoral pictures. It could be the case that individuals with lower IQs are less capable of separating negative situations from immoral situations or that they tend to overinterpret the information shown to them (e.g., if people are shouting, someone must have done something wrong).

The lack of amygdala activity across both groups for the immoral > nonmoral contrast as well as the lack of relation between amygdala activity and psychopathy scores may indicate that amygdala dysfunction in psychopathy might not be specifically relevant to moral evaluations, nor to moral evaluation in general. There is strong evidence for amygdala dysfunction in emotion processing in psychopathy [e.g., (30–33)], but the evidence for a relation with moral processing is less clear. For example, negative relations between amygdala activity and psychopathy have mostly been found in immoral + nonmoral > neutral, immoral > neutral, and nonmoral > neutral contrasts, but not in immoral > nonmoral contrasts (21, 22). This would also explain why (19) did find an effect in the amygdala as in their analysis they specifically contrasted “moral personal emotion-provoking” and “moral impersonal less emotional” dilemmas. Additionally, a recent and specific meta-analysis on moral processing found no evidence for amygdala involvement (16) in contrast to an older meta-analysis (34).

In the vmPFC, we observed a positive relationship between psychopathic traits and brain activity during moral evaluation, indicating that young adults high in psychopathic traits show a greater increase in vmPFC activity during moral evaluation than young adults low in psychopathic traits. One possible explanation is that individuals high in psychopathic traits have to recruit this region to a greater extent in order to reach a normal level of moral evaluation. In fact, studies have shown increased prefrontal brain activity in criminal psychopaths during emotion processing (32), increased dorsolateral prefrontal brain activity in community volunteers with high psychopathic traits during moral processing (35), and the affective trait of psychopathy has been found to be positively related to frontal brain activity when viewing fearful faces (31). Likewise, in a study in healthy participants, participants with lower moral judgment competence showed increased vmPFC activity during a moral evaluation task compared to participants with higher moral judgment competence scores. In this study, participants performing worse showed increased rather than decreased brain activity (36). That such a mechanism of overcompensation is present in our sample and not in most studies on psychopathic traits and moral evaluation may be due to the specific age range of our sample or its low IQ. Whereas in other studies participants usually have average to high IQs, the average IQ within our sample is low and the variation is limited, it may be the case that a combination of high psychopathic traits and low IQ required multi-problem young adults to engage more brain activity in order to perform the task. Another explanation for the association is that as the vmPFC is a large brain area, distinct areas within the vmPFC may perform different functions when making moral evaluations. Therefore, generalizing activity from specific clusters across the vmPFC may not be prudent and future research disentangling distinct areas functioning within the vmPFC is needed to elucidate this.

In addition to the effects observed in the vmPFC, we found positive relations of similar strength between psychopathic traits and brain activity during moral evaluating in the left CG and the left STG. In accordance with our vmPFC finding, the callous-unemotional affective factor of psychopathy accounted for these observations. A previous studies also reported a negative correlation between moral CG activity and psychopathic traits (19) but no correlations between moral STG activity and psychopathic traits. However, a negative correlation in the STG has been observed using an immoral + nonmoral > neutral contrast (22). As we included the CG and STG as ROIs, our analysis is more sensitive to effects in these areas and other studies may have missed these. Our results suggest that most of the brain regions associated with moral evaluating behave aberrantly in psychopathy, rather than the vmPFC and possibly amygdala specifically.

As the effects we observed are all specific to the callous-unemotional affective trait of psychopathy, this is likely the main factor related to moral evaluation in our population. This does not seem surprising since moral evaluating requires empathy to assess a situation, whereas for example impulsivity and grandiosity may be more relevant when deciding what to do. It is possible that deficits in moral evaluation are driven by the callous-unemotional affective trait, whereas deficits in moral response decisions may also be driven by the impulsive and irresponsible behavioral, and grandiose-manipulative interpersonal traits. One study has shown psychopathic traits in a healthy sample to be related behaviorally to moral decisions, but not moral judgment (37). It would be valuable for future research to delve into the neurobiological differences between moral evaluations and moral response decisions, and investigate whether distinct psychopathic traits are differentially related to these processes.

A limiting factor in comparing our study to others is the fact that we used the YPI, which is a self-report questionnaire, to assess psychopathy rather than the more extensive PCL-R or PCL-YV (38). The YPI has good convergent validity (25) and the YPI and the PCL-YV correlate with moderate strength (39), so it seems unlikely the positive relation between psychopathy and brain activity during moral evaluations is due to the use of a self-report measure. However, other research has suggested that psychopathy subscales specifically are not interchangeable between different assessment instruments (40), so caution is warranted. Another limitation is that we did not use urine screens or breathalyzer to objectively assess whether participants adhered to the instruction to refrain from substance use prior to the MRI session, all participants reported adhering to the instructions.

In conclusion, we found brain activation related to moral evaluation in the STG, in several distinct areas of the vmPFC, in the precuneus, the parahippocampal gyrus, the middle occipital gyrus (MOG), and in the cerebellum in a group of multi-problem young adults and healthy controls; we found that brain activity in the left vmPFC, left STG, and left CG was associated with the affective callous-unemotional traits of psychopathy; and we found that IQ, but not psychopathic traits are related to the moral evaluations of participants. Our results add to the evidence that brain dysfunction underlies psychopathic traits in moral evaluation and suggest that most of the brain regions associated with moral evaluating are affected in young adults with higher levels of psychopathy, specifically on the affective callous-unemotional dimension, rather than the vmPFC and possibly amygdala alone.

## Author contributions

JZ, RM, HT, and AP contributed to the study design; JZ and RM collected the fMRI data; JZ performed statistical analysis and wrote the first draft of the manuscript; RM, FB, M-JL, and LvD wrote sections of the manuscript; all authors contributed to manuscript revision, read, and approved the submitted version.

### Conflict of interest statement

The authors declare that the research was conducted in the absence of any commercial or financial relationships that could be construed as a potential conflict of interest.
